# Effects of a 2-km Swim on Markers of Cycling Performance in Elite Age-Group Triathletes

**DOI:** 10.3390/sports7040082

**Published:** 2019-04-05

**Authors:** Jeffrey Rothschild, George H. Crocker

**Affiliations:** 1 TriFit Performance Center, Santa Monica, CA 90404, USA; 2 Nutrition and Functional Medicine Program, University of Western States, Portland, OR 97230, USA; 3 School of Kinesiology and Nutritional Science, California State University Los Angeles, Los Angeles, CA 90032, USA; gcrocke@calstatela.edu

**Keywords:** triathlon, cycling, swimming, lactate, VO_2max_, Ironman

## Abstract

The purpose of this study was to examine the effects of a 2-km swim on markers of subsequent cycling performance in well-trained, age-group triathletes. Fifteen participants (10 males, five females, 38.3 ± 8.4 years) performed two progressive cycling tests between two and ten days apart, one of which was immediately following a 2-km swim (33.7 ± 4.1 min). Cycling power at 4-mM blood lactate concentration decreased after swimming by an average of 3.8% (*p* = 0.03, 95% CI −7.7, 0.2%), while heart rate during submaximal cycling (220 W for males, 150 W for females) increased by an average of 4.0% (*p* = 0.02, 95% CI 1.7, 9.7%), compared to cycling without prior swimming. Maximal oxygen consumption decreased by an average of 4.0% (*p* = 0.01, 95% CI −6.5, −1.4%), and peak power decreased by an average of 4.5% (*p <* 0.01, 95% CI −7.3, −2.3%) after swimming, compared to cycling without prior swimming. Results from this study suggest that markers of submaximal and maximal cycling are impaired following a 2-km swim.

## 1. Introduction

The sport of triathlon combines three disciplines (swimming, cycling, and running) into a single race, with competitions lasting from less than 2 h for sprint-distance to between 8 and 16 h for full-distance (Ironman™) triathlons [1,2]. The three main factors that predict endurance sport performance are maximal oxygen consumption (VO_2max_), lactate threshold, and movement economy [3]. Movement economy is determined by the amount of oxygen needed to produce a given running speed, swimming velocity, or cycling power [3]. Performance VO_2_ has been defined as the rate of oxygen consumption (VO_2_) that can be sustained for a given race duration and is dependent on VO_2max_ and the lactate threshold [3]. These variables are commonly measured by triathletes and are important in triathlon performance [4,5].

Many triathletes undergo metabolic and lactate testing in order to monitor training progress and determine cycling power and heart rate (HR) zones, and these data may also predict triathlon performance [6]. For example, cycling performance in an Olympic-distance triathlon is correlated with both VO_2max_ and power at the lactate threshold in recreational triathletes [7]. In addition, peak workload attained during an incremental test to fatigue has been found to correlate with VO_2max_ and 20-km time-trial performance in well-trained cyclists [8]. In recreational triathletes, overall triathlon finishing time can be predicted from VO_2max_ and VO_2_ at the ventilatory threshold during an incremental cycling test [5].

Because triathletes must cycle after the swimming portion of the race, it is important to understand how cycling power and HR may be affected by prior swimming. Kreider et al. [9] found that an 800-m swim decreased mean cycling power and VO_2_ during a simulated triathlon (40-km bike, 10-km run) compared to without prior swimming. Blood lactate concentration was higher following a 1500-m swim in well-trained triathletes cycling at 75% of peak power, compared to a cycling-only trial [10]. In contrast, Laursen et al. [11] found no significant performance effects of a 3-km swim on subsequent 3-h cycling time-trial performance in well-trained triathletes. Therefore, it is possible that the effects of swimming on subsequent cycling performance may be dependent on the intensity and/or duration of the swim. However, there is a paucity of data investigating how the swimming portion of a triathlon affects physiological variables such as VO_2max_, cycling economy, and lactate profiles during cycling. The purpose of this study was to determine the effects of a 2-km swim performed prior to a progressive cycling test on VO_2max_, peak power, power at the 4-mM lactate threshold, and cycling economy. We hypothesized that all of these four measurements would decrease following a 2-km swim compared to the same test without prior swimming and that subjects would have a higher HR during submaximal cycling and at the 4-mM lactate threshold. Secondary outcomes were to determine correlations between markers of swimming intensity and subsequent changes in cycling performance.

## 2. Materials and Methods

### 2.1. Experimental Protocol

This study determined how a 2-km swim affects markers of cycling performance by having a group of well-trained triathletes report to the lab on two separate days, between two and ten days apart. On both days, subjects underwent both submaximal and maximal graded cycling tests. On one of the two days, subjects performed a 2-km swim at half-distance triathlon intensity before the cycling tests. Testing days were counterbalanced, so half of the participants performed the swim-bike testing first, whereas the other half did the bike-only testing first. For diet standardization, participants kept food journals and recorded their 24-h intake prior to the first testing session and repeated it before the second testing session.

### 2.2. Participants

Fifteen age-group triathletes (10 men, 5 women) with a mean VO_2max_ of 57.7 ± 6.3 mL·kg^−1^·min^−1^ participated in this study (Table 1). Cycling data for one male were excluded from the analysis due to technical difficulties with the testing equipment. Inclusion criteria were having at least one of the following within the past two years: (1) finishing time under 10 h and 30 min in a full-distance (3.9-km swim, 180-km bike, 42.2-km run) triathlon; (2) finishing time under 5 h for a half-distance (1.9-km swim, 90-km bike, 21.1-km run) triathlon; or (3) qualifying for the full or half-distance world championships in either professional or age-group categories. Participants were recruited by word of mouth among local triathletes, along with an email announcement to a local triathlon club. Participants were given a detailed explanation of all tests and data that would be collected prior to obtaining written informed consent and before the start of testing. Ethics committee approval was provided by the University of Western States (IRB20110127).

### 2.3. Swimming Protocol

For the swim-bike session, the participants began by swimming 2012 m in a 25-yard (22.86-m) indoor swimming pool (88 lengths). A researcher standing on the pool deck monitored swim distance. Pool temperature was 28 °C for all subjects. Participants were instructed to swim at a perceived effort equivalent to their half-distance triathlon race pace. Heart rate while swimming was measured continuously with a wearable HR monitor (Garmin 920XT, HR-swim, Garmin, Olathe, KS, USA) and was averaged to get a single value for the entire swim. Additionally, average HR and swim velocity during Laps 22, 44, 66, and 88 were analyzed, representing segments at 25%, 50%, 75%, and 100% of the total swimming distance, respectively. Mean swimming HR was recorded and reported as the percentage of each individual’s maximal HR observed during the maximal cycling test (HR_swim_). However, due to technical difficulties with the HR strap, we were only able to analyze HR data for 13 swimmers. Blood samples (0.2 µL) were collected from the left index finger immediately following the swim and analyzed for lactate concentration using a portable blood lactate analyzer (Lactate Pro 2, Carlton, Australia). Participants promptly towel-dried, changed into their cycling clothing, and were weighed immediately prior to cycling. Hydration changes during the swim were assessed by weighing subjects in their cycling clothing before and after swimming. The time between the completion of the swim to start of the cycling warm-up protocol was approximately 10 min for all subjects. Body mass and body composition were assessed using an eight-point bioelectrical impedance analyzer (mBCA 514, seca Ltd., Hamburg, Germany).

### 2.4. Cycling Protocol

Cycling was performed on an electronically-braked indoor cycle trainer (CompuTrainer^®^ RacerMate Inc., Seattle, WA, USA), which allows participants to use their own bicycle and pedal at a self-selected cadence while eliciting a constant power. All cycling sessions were conducted in standard laboratory conditions (18–22 °C, 40–50% relative humidity), and subjects were fan-cooled (Honeywell HT-900, Palatine, IL, USA) while cycling.

Following a 10-min warm-up (<80 W), subjects underwent submaximal and maximal graded exercise tests. The submaximal test began at 150 W for men and 80 W for women, and increased by 35 W every 4 min for both sexes. The submaximal test was stopped at the end of the stage that produced a blood lactate concentration of >4 mM, based on a protocol used to study an elite cyclist [12]. Subjects were given a 10-min active recovery, during which the athlete consumed water ad libitum and cycled at 50 W, before starting the maximal test.

The maximal exercise test started at 100 W for males and 70 W for females and increased by 30 W every min until volitional fatigue. Participants were instructed to maintain a cadence of at least 70 RPM. The test was terminated when the participant was unable to maintain a cadence of >70 rpm, despite verbal encouragement. The mean (± SD) duration for the submaximal testing was 18.0 ± 3.0 min and 9.9 ± 1.8 min for the maximal testing. These were performed as two separate tests because VO_2max_ values should be obtained with a graded exercise test between 8 and 12 min, as the highest VO_2_ values obtained during extended tests may be underestimated [13].

Expired gases were collected and analyzed for assessment of VO_2_ and the rate of CO_2_ production using a metabolic cart (CardioCoachCO_2_, Korr Medical Technologies, Inc., Salt Lake City, UT, USA). Subjects breathed into and out of a silicone oronasal facemask connected to a two-way non-rebreathing valve (Hans Rudolph, Inc., Shawnee, KS, USA). The expiratory port of the respiratory valve was connected to a 1.8-m breathing tube that connected to a 5-L mixing chamber. Heart rate was measured using a chest strap (Polar T31, Polar, Inc., Kempele, Finland) that transmitted to the metabolic cart. Heart rate and VO_2_ were measured continuously.

Expired gas data were averaged across 15-s intervals using the internal gas analysis software (Korr Medical Technologies, Inc., Salt Lake City, UT, USA) before exporting for further analysis. Steady-state VO_2_ for each stage of the submaximal test was calculated as the average of the last two measurements (final 30 s). Cycling economy was calculated as the power (W) at a submaximal steady-state workload (220 W for men; 150 W for women) divided by VO_2_ at that power (i.e., W·L^−1^·min) [14]. The exercise intensity for these measurements was ~55% of peak power for both sexes (56.0 ± 3.8% for males; 54.0 ± 9.6% for females).

Blood samples (0.2 µL) were collected from the left index finger during the final 15 s of each stage of the submaximal aerobic testing. Blood lactate concentrations, power output, and HR were input into validated software (Lactate-E) [15], which subsequently calculated the predicted power and HR at a lactate concentration of 4 mM. Due to the variations observed in lactate threshold among different calculation methods [16] and because the shape of the lactate curves were likely to be influenced by the elevated lactate levels after swimming, a fixed point of 4 mM was used for comparison across tests [17].

Peak power was determined by the workload in the last completed stage plus the workload relative to the time spent in the last incomplete stage (peak power = power for completed stage + 30 × (seconds at uncompleted stage/60)). Peak power relative to body mass was calculated using the body mass measurement taken immediately preceding the cycling testing (i.e., after the swim during the swim-bike trial). VO_2max_ was determined by the highest 30-s average collected during the maximal test.

### 2.5. Statistical Analyses

Data are reported as the means ± standard deviations. Measurements from the swim-bike and bike-only trials were compared using paired *t*-tests. An independent samples *t*-test was used to make comparisons between sexes. Pearson’s correlations were used to determine relationships between and within measures of swimming intensity and changes in key markers of cycling performance. All tests were performed with the significance set at *p* < 0.05. All statistical tests were performed using SPSS Version 24 software.

## 3. Results

### 3.1. Submaximal Aerobic Profiles

**Figure 1:**
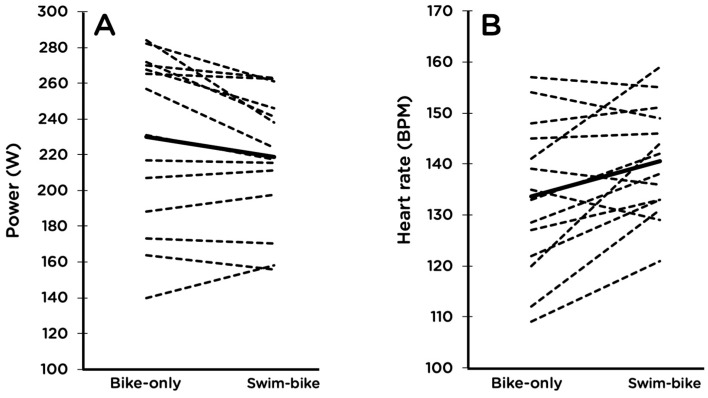
Power at 4 mM blood lactate concentration (**A**) and heart rate at 220 W for males and 150 W for females (**B**) with and without a prior 2-km swim. For each panel, the solid line denotes the group mean and the dashed lines are individual responses. Group means were significantly different from each other for both endpoints.

Power at 4-mM blood lactate concentration decreased by 3.8% (*p* = 0.03, 95% CI −7.7, 0.2), and HR at 220 W for men and 150 W for women increased by 4.0% (*p* = 0.02, 95% CI 1.7, 9.7) after swimming compared to without prior swimming (Figure 1). There were no changes in HR at 4 mM lactate or cycling economy (Table 2). Increases in HR at 220 W for men and 150 W for women after swimming were correlated with decreases in power at 4 mM (r = −0.64, *p* = 0.01).

### 3.2. Maximal Aerobic Profiles

**Figure 2:**
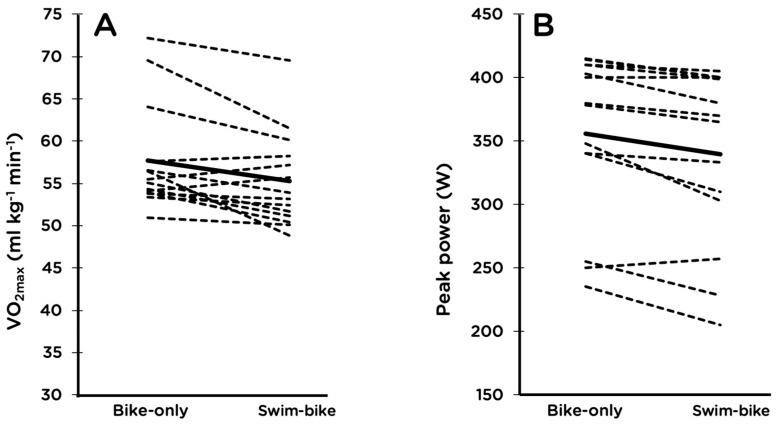
Maximal oxygen consumption (VO_2max_; **A**) and peak power (**B**) during an incremental cycling test to exhaustion with and without a prior 2-km swim. For each panel, the solid line denotes the group mean and the dashed lines are individual responses. Group means were significantly different from each other for both variables.

Cycling VO_2max_ decreased by 4.0% (*p* = 0.01, 95% CI −6.5, −1.4), and peak power decreased by 4.8% (*p* = 0.0008, 95% CI −7.3, −2.3) after the 2-km swim compared to without prior swimming (Figure 2). Maximal HR did not differ between the two trials (Table 2).

### 3.3. Swimming Intensity

**Figure 3:**
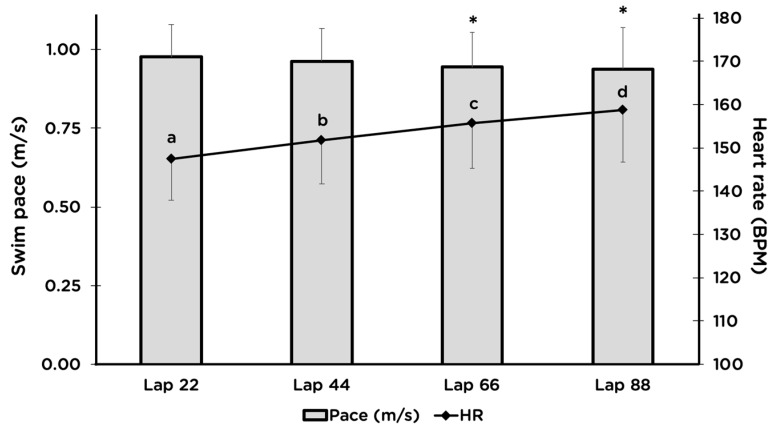
Average swimming pace (bars) and heart rate (line) during Laps 22, 44, 66, and 88 of an 88-lap swim in a 22.86-m (25-yd) pool. Values are the mean ± SD. Heart rate differed significantly for all four laps. * denotes significantly slower swim speed than Lap 22 (n = 13).

Average 2-km swimming time was 33.7 ± 4.1 min, which equates to a pace of 1:41 per 100 m. Swimming HR was 151 ± 9 BPM, which was 88.9 ± 3.9% of HR_max_ from the maximal cycling test. Blood lactate concentration immediately after swimming was 6.5 ± 2.3 mM, and there was a decrease in body mass during swimming of 1.0 ± 0.4%. Swimming HR rose steadily throughout the swim, despite a small, but statistically-significant decrease in swim speed (Figure 3).

**Figure 4:**
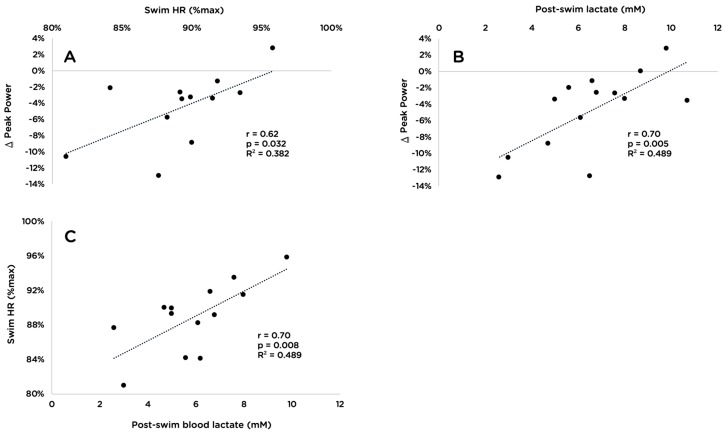
Relationship between changes in peak power attained during an incremental cycling test to exhaustion and swimming intensity as a percentage of maximal heart rate (**A**; n = 12) and blood lactate at the completion of the swim (**B**; n = 14); and between swimming intensity as a percentage of maximal heart rate and blood lactate at the completion of the swim (**C**; n = 13).

Swimming HR as a percentage of HR_max_ was positively correlated with both changes in peak power (r = 0.62, *p* = 0.03; Figure 4A) and post-swim lactate concentration (r = 0.70, *p* = 0.01, Figure 4C). Post-swim lactate concentration and changes in peak power were also positively correlated (r = 0.70, *p* = 0.01, Figure 4B). Additionally, individuals who lost a greater percentage of body mass while swimming tended to have a smaller decrease in peak power (r = −0.53, *p* = 0.052).

## 4. Discussion

The main findings of this study were that a 2-km swim prior to cycling decreased VO_2max_, peak power and power at 4 mM lactate concentration by 4.0%, 4.8%, and 3.8%, respectively, and increased HR during submaximal cycling by 4.0%. These findings support our hypothesis that VO_2max_, peak power and power at the lactate threshold would decrease and submaximal HR would increase when cycling after a 2-km swim compared to without prior swimming. However, we also hypothesized that subjects would have a higher HR at their lactate threshold and that cycling economy would decrease after swimming, and this portion of our hypothesis was not supported. In addition, HR at 4 mM lactate concentration and peak HR were similar for both conditions.

There are several possible mechanisms for the observed impairments in cycling following swimming. Muscle and/or liver glycogen depletion may be one mechanism. Glycogen depletion prior to a graded exercise test can cause the lactate threshold to be reached at lower power [18,19], though it is unclear how depletion of primarily upper-body intramuscular glycogen from swimming may impact subsequent lower-body cycling exercise. Many triathletes consume carbohydrates at the swim-to-bike transition, but we chose not to provide any food or drink besides water in order to isolate the effects of the 2-km swim from exogenous carbohydrate ingestion. However, it is unlikely that lower-body muscle glycogen was significantly different between trials as there were no differences in respiratory exchange ratio (RER) during submaximal cycling (220 W for males, 150 W for females, 0.87, Table 2). Differences in RER could be expected with different starting concentrations of muscle glycogen, indicating a greater reliance on fat metabolism and a lower RER [20]. The average between-day difference in body mass for the athletes in this study was 0.3 ± 0.6% (*p* = 0.08), suggesting similar starting levels of carbohydrate and water storage [21].

Dehydration incurred while swimming is another potential mechanism for the impairments in cycling capacity following the swim. Our subjects lost 1.0 ± 0.4% (range −0.4 to −1.4%) of their body mass during the swim, suggesting at least mild levels of hypohydration. This is equivalent to a sweat rate of 1.3 L·h^−1^, which is similar to sweat rates reported in one swimming study [22], but higher than another [23]. Moquin and Mazzeo [19] observed a 12% decrease in power at lactate threshold after ~2.6% body mass loss via fluid restriction compared with a euhydrated state. Similarly, Green et al. [24] observed that 2% dehydration increased HR and blood lactate during steady-state cycling compared to cycling in a euhydrated state. Kenefick et al. [25] found that the lactate threshold occurred at a lower exercise intensity after 4% exercise-induced hypohydration. In accordance with previous research, dehydration appears likely to have contributed to the 3.8% decrease in power at 4 mM blood lactate during cycling following the 2-km swim, despite the lack of negative impact on peak power. 

Elevations in core body temperature are likely to have also played a role in the cycling impairment after swimming, although this was not directly measured in our study. As core temperature increases, there is a shunting of the blood to the cutaneous vessels to dissipate heat, reducing stroke volume and increasing HR to maintain cardiac output [26,27]. Elevated core temperature could also lead to an increased rate of anaerobic glycolysis, resulting in an increased lactate accumulation [28]. Kreider et al. [9] reported a 0.72 °C increase in core temperature following an 800-m swim (~17 min) in a 23 °C pool, and Costill et al. [29] reported increases in core temperature (between 0.24 and 0.72 °C) during 20 min of submaximal swimming that were related to the water temperature within the range of 17–34 °C. Considering that the pool used in the present study was maintained at 28 °C and subjects swam 2012 m, we can speculate that subjects incurred at least some degree of hyperthermia while swimming. Taken together, it is likely that the elevated HR and blood lactate concentrations when cycling after swimming may have been related to dehydration and an elevated core temperature.

Limited data exist looking at the swim-to-bike portion of triathlons. Other studies have examined performance outcomes, blood lactate parameters, and cycling economy [9,10,11,30,31]. To our knowledge, the present study is the first investigation of the effects of swimming prior to submaximal and maximal incremental cycling tests. We found a ~4% decrease in power at the 4 mM lactate threshold after swimming and no change in cycling economy. Similar to our study, other research in well-trained triathletes has found higher levels of blood lactate and no change in cycling economy while cycling at 75% of peak power following a 1500-m swim, compared to cycling without prior swimming [10]. A 17% decrease in cycling power during a 40-km cycling time-trial and a 6% increase in VO_2_ following an 800-m swim have also been reported [9]. In contrast, no significant effects of a 3-km swim were found on subsequent 3-h cycling time-trial performance in well-trained triathletes [11]. However, in that study, mean power output was 10 W lower after the swim trial, and when considering their small sample size (*N* = 8), it is possible that the study lacked the statistical power to detect the 10-W difference between groups. The differences between the present study and previous research are likely attributable to the differences in test duration, as longer-duration swim and cycling tests would be performed at a lower intensity and have less lactate accumulation. For example, blood lactate concentrations were 8.0 mM after a 750-m swim at triathlon race-pace intensity [32], 6.9 mM after a 1.5-km swim [10], 6.6 mM immediately after the 2-km swim in the present study, and 5.5 mM after a 3-km swim [11]. However, despite the observed impairments in markers of cycling capacity in our study, the lack of any performance testing (e.g., time-trial or simulated triathlon) makes direct comparisons between studies difficult.

Subjects in our study were elite age-group triathletes, with the majority (67%) having qualified for at least one full- or half-distance world championship event and the others achieving finishing times below 10.5 h in a full-distance or 5 h in a half-distance triathlon. Mean VO_2max_ values for the men in this study were lower than the 69–75 mL·kg^−1^·min^−1^ measured in elite male triathletes [33,34,35], but similar to age-group triathletes (57.4 mL·kg^−1^·min^−1^) competing at the Ironman™ World Championships [36]. The females in this study had VO_2max_ values of 59.3 ± 7.1 mL·kg^−1^·min^−1^, which are similar to the 60–61 mL·kg^−1^·min^−1^ reported in elite female triathletes [6,33]. Peak power in our study (394 ± 25 W for males, 286 ± 54 W for females) was similar to values reported for elite triathletes of both sexes [6,33]. Cycling economy in our study was 69–73 W·L^−1^ min, which is similar, but on the lower end of what has been reported in elite male and female triathletes (72–80 W·L^−1^ min) [33,34]. 

Testing for this study was performed during off-season months of the triathlon season (October–December). Although the athletes were still training, it is likely that some of the participants had experienced some amount of detraining compared with their racing fitness levels [37]. Body fat percentages for the men and women in the present study were 15.4 ± 4.1% and 18.1 ± 4.1%, respectively. Male body fat percentages were higher than values reported for elite male triathletes (7–10%), while female body fat percentages were in the range of elite female triathletes (12–20%) [6,36]. These higher values may also be related to off-season detraining, as a number of the participants reported weighing 2–6 kg more than their typical racing weight. Furthermore, athletes in this study ranged from 29–56 years old. Despite continued training, a gradual increase in body fat percentage is observed with aging [38], along with decreases in VO_2max_ [39]. Indeed, slower race times in long-course triathlons have been observed after the age of 30 for women and 35 for men [1]. Taken together, participants in this study displayed levels of fitness that would be expected from their status as high-level age-group competitors, though the results of this study may not be generalizable to professional or recreational triathletes.

The swim portion of a long-course triathlon is either 1.9-km (half-distance) or 3.8-km (full-distance), which takes approximately 25–35 min and 50–75 min to complete, respectively. We chose a distance of 2-km for this study because many triathletes only compete in the half-distance races. Participants were instructed to swim at a perceived exertion similar to how they would swim in a half-distance triathlon. Average HR during the swim was 151 ± 9 beats per minute, which corresponded to ~89% of the HR_max_ achieved during cycling. However, it has been shown that HR_max_ measured while swimming is lower than the HR_max_ attained during cycling and running, due to the effects of a horizontal body position and being submerged underwater, leading to a greater venous return and a higher stroke volume [40,41,42,43]. Thus, it is likely that these athletes were swimming at an even greater percentage of their swimming HR_max_. Furthermore, HR drifted upward for the duration of the swim despite slower swim velocity during Laps 66 and 88 (Figure 3). This can be suggestive of cardiovascular drift, which results largely from dehydration and concomitant hyperthermia [44]. Another possible explanation for the increasing HR is an increase in the VO_2_ slow component, which results from exercise performed above the lactate threshold and represents a decrease in skeletal muscle contractile efficiency [45]. Peeling et al. [35] compared three different swim intensities on subsequent cycling and overall sprint-distance triathlon performance and found that cycling efficiency was higher after a lower-intensity swim than it was after a maximal-intensity swim, which translated into faster cycling and faster overall sprint-triathlon performance. Post-swim lactate in our study was 6.5 mM, which is similar to the moderate-intensity swim in the Peeling et al. study (6.7 mM) that resulted in no differences in cycling efficiency or triathlon time compared to the higher- or lower-intensity trials [35].

Due to the controlled nature of laboratory testing, this study was unable to replicate the dynamics of an open-water swimming race. For example, drafting is used as a tactic during the swim portion of a triathlon, and increased time-trial power and cycling efficiency have been observed after swimming in a drafting position compared with swimming in isolation [30,32]. Pacing when swimming solo in a pool is also likely to be more constant compared to racing in open water where triathletes change speeds based on the competitors around them. Future studies should investigate how different swim pacing strategies affect cycling time-trial performance and different physiologic measurements on a graded exercise test. It would also be valuable for field-based studies to obtain data for heart rate and blood lactate upon completion of the swim portion of a triathlon.

The secondary outcome of this study was to determine the relationships between and within measures of swimming intensity and changes in key markers of cycling performance. There was a strong positive correlation between two main indices of swimming intensity: HR (as a percentage of each individual’s HR_max_) and post-swim blood lactate levels (Figure 4C). We also found positive correlations between both measures of swimming intensity and changes in peak power (Figure 4A,B). Participants who completed the swim with the highest blood lactate and highest HR had the smallest decreases in peak power during the maximal cycling test. Consistent with this observation, there was a tendency for individuals who lost a greater percentage of body mass while swimming to also have a smaller decrease in peak power (*r* = −0.53, *p* = 0.052). While this finding may seem counterintuitive, it is in accordance with previous research showing that following high-intensity exercise, there is a shift toward greater aerobic energy contribution [46] and an increase in perimaximal exercise performance [47]. Future research should examine the effects of prior swimming on the power–time relationship during subsequent cycling.

Translating the results of the present study is challenging, as we did not have a direct measure of performance. We chose to have the subjects perform submaximal and maximal graded exercise tests to determine physiological endpoints, rather than time-trial performance [9,11], which represents both the novelty and primary limitation of the study. Thus, caution must be used when extrapolating these results to triathlon performance. For the lactate threshold, we chose 4 mM as our reference point because the shapes of the lactate curves may have been influenced by the elevated lactate concentration after swimming. However, power at 4 mM may overestimate the lactate threshold compared to other commonly-used methods for estimating the lactate threshold [16]. This study was also limited by its lack of ecological validity, as a solo pool swim is different from open-water racing conditions. In addition, athletes have shorter swim-to-bike transitions, consume carbohydrates during the transition and on the bike, and do not have a warm-up period between the swim and the bike.

## 5. Conclusions

This study demonstrated that a 2-km swim prior to cycling decreased VO_2max_, peak power, and power at the lactate threshold by ~4–5%, while also increasing HR during submaximal cycling, in elite age-group triathletes. These findings are applicable for triathletes who undergo metabolic and lactate testing in order to monitor training progress and determine cycling power and heart rate zones, and who must cycle (and then run) after swimming as part of their race. It would be beneficial for triathletes to perform cycling tests following a swim that approximate their race distance and intensity, in order to determine target HR and power zones at which to train. Furthermore, athletes and coaches should consider different swim-pacing strategies to determine its effects on cycling and overall triathlon performance.

## Figures and Tables

**Table 1:** Subject characteristics and personal-best triathlon times. Data are presented as the mean ± SD. † denotes significantly different from males (*p* < 0.05). Data are for 15 subjects (10 men, 5 women), unless otherwise noted.

Subjects	Age (year)	Height (m)	Body Mass (kg)	BMI (kg/m^2^)	Body Fat (%)	Half-Distance Personal Best (h) ^a^	Full-Distance Personal Best (h) ^b^
All	38.3 ± 8.4	1.76 ± 0.12	69.5 ± 13.7	22.4 ± 2.1	15.6 ± 4.5	4.69 ± 0.26	10.15 ± 0.49
Males	37.2 ± 8.1	1.82 ± 0.08	77.1 ± 7.0	23.5 ± 1.3	14.4 ± 4.1	4.62 ± 0.22	10.07 ± 0.62
Females	40.6 ± 9.4	1.63 ± 0.10 †	54.1 ± 10.3 †	20.1 ± 1.4 †	18.1 ± 4.1	4.84 ± 0.30	10.27 ± 0.17

^a^ n = 13 (9 M, 4 F), ^b^ n = 9 (5 M, 4 F).

**Table 2:** Comparison of markers of submaximal and maximal cycling for bike-only and swim-bike trials. Submaximal endpoints were power and heart rate (HR) at 4 mM blood lactate concentration and cycling economy and HR at a predetermined workload of 220 W for males and 150 W for females (~55% peak power). Maximal endpoints were respiratory exchange ratio (RER), maximal rate of oxygen consumption (VO_2max_), maximal HR (HR_max_), peak power and relative peak power. Data are presented as the mean SD. * indicates a significant difference between trials (*p* < 0.05).

Trial	Power at 4 mM (W)	HR at 4 mM (BPM)	Economy at 220 W (m) or 150 W (f) (W/L O_2_)	HR at 220 W (m) or 150 W (f) (BPM)	RER at 220 W (m) or 150 W (f)	VO_2max_ (mL·kg^−1^·min^−1^)	HR_max_ (BPM)	Peak Power (W)	Relative Peak Power (W·kg^−1^)
Bike-only	230 ± 48	147 ± 8	70.3 ± 5.3	134 ± 15	0.87 ± 0.4	57.7 ± 6.3	171 ± 9	356 ± 65	5.15 ± 0.46
Swim-bike	219 ± 37	148 ± 9	71.9 ± 3.8	141 ± 11	0.87 ± 0.3	55.3 ± 5.7	168 ± 10	340 ± 69	4.94 ± 0.44
Mean % change [95% CI]	−3.8 [−7.7, 0.2]	2.5 [−1.4, 3.6]	3.3 [−0.7, 5.9]	4.0 [1.7, 9.7]	−0.8 [−2.7, 1.1]	−4.0 [−6.5, −1.4]	−1.4 [−2.8, 0.05]	−4.8 [−7.3, −2.3]	−4.0 [−6.7, −1.3]
*p-*value	0.03 *	0.43	0.18	0.02 *	0.38	0.01 *	0.058	<0.01 *	0.01 *
